# Blunt penetration technique for treatment of a completely obstructed anastomosis after rectal resection: a case report

**DOI:** 10.1186/1752-1947-8-236

**Published:** 2014-06-27

**Authors:** Keiichi Yazawa, Daisuke Morioka, Chizuru Matsumoto, Yasuhiko Miura, Shinji Togo

**Affiliations:** 1 Department of Surgery, Yokohama Ekisaikai Hospital, 1-2 Yamada-cho, Naka-ku, Yokohama 231-0036, Japan

**Keywords:** Colorectal anastomosis, Complete obstruction of intestinal anastomosis, Endoscopic treatment

## Abstract

**Introduction:**

We present a case of completely obstructed anastomosis after rectal resection which was nonsurgically and successfully treated with a blunt penetration technique using a commonly used device for transanal ileus drainage. The technique we used in this case has not been previously reported.

**Case presentation:**

A 79-year-old Japanese man underwent redo rectal resection for completely separated anastomosis which was caused by anastomotic leakage after a sigmoidectomy performed 3 years previously that was remedied by diverging ileostomy. Immediately after the redo surgery, fluoroscopy showed good passage through the colorectal anastomosis but no anastomotic leakage. However, fluoroscopy and colonoscopy prior to the ileostomy takedown showed complete obstruction of the anastomosis. Unlike usual anastomotic strictures, the lumen between colon oral and rectum anal to the anastomosis was completely discontinued by a membranous structure. Therefore, a conventional balloon dilatation technique was unsuitable for this condition. We applied a blunt penetration technique using a commercially available device designed as a transanal drainage system for obstructing colorectal cancer to restore the continuity between the colon oral and rectum anal to the anastomosis. After restoring the continuity, we performed conventional balloon dilatation for the anastomosis and successfully treated the anastomotic obstruction. Subsequently, the patient underwent ileostomy takedown and is currently doing well 12 months after the ileostomy takedown.

**Conclusions:**

The penetration technique we applied is easy and less stressful to adopt because it does not require usage of materials specialized for other particular purposes. Furthermore, we believe that this technique is superior in safety to other reported methods for this condition even if applied in the wrong direction because this technique does not utilize electrocision or sharp needle puncture.

## Introduction

Complete obstruction of colorectal anastomosis after rectal resection is rare but has been reported in certain populations [1]. Because of its rareness, the treatment strategy for this condition depends on each individual situation [2-5]. Lefevre *et al.* reported from their study concerning 32 cases of redo surgery for failed colorectal or coloanal anastomosis and concluded that redo surgery is feasible treatment for this condition [1]. However, the morbidity rate after the redo surgery was reported to be considerably high with 46% classified as having a Clavien–Dindo II-IV complication [1,6]. Therefore, nonsurgical treatment for this condition was tried and reported to be successful in some cases [2-5]. Because of its rare occurrence, there is no consensus for nonsurgical treatment of this refractory condition. Thus, reporting new cases is useful for sharing valid experiences. Here, we present a case of completely obstructed anastomosis after rectal resection which was nonsurgically and successfully treated with blunt penetration technique using a commercially and commonly available device for transanal ileus drainage.

## Case presentation

**Figure 1:**
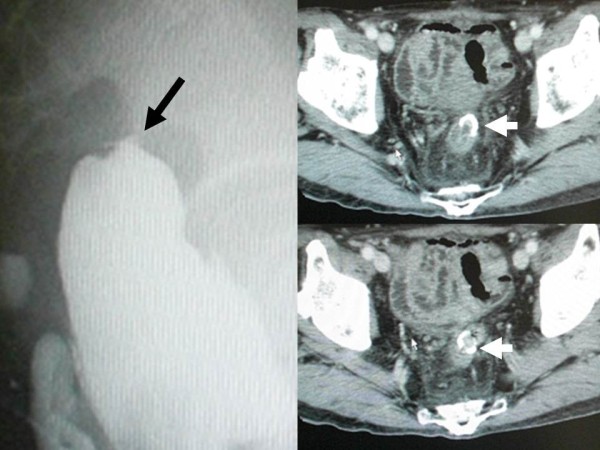
**Findings of the fluoroscopy and abdominal computed tomography 6 months after the redo surgery.** Fluoroscopy showed that anastomosis of the redo rectal resection was completely occluded (left, black arrow). Computed tomography showed that well-enhanced tissue was present inside the anastomotic circular stapler ring (upper and lower right, white arrows), suggesting that patency of the anastomosis was discontinued by the thick membranous web.

**Figure 2:**
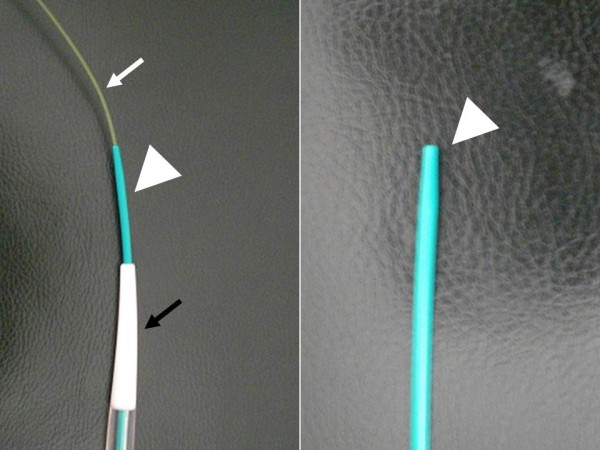
**Appearance of the transanal ileus drainage system (Transanal Ileus Tube®; Create Medic Co., Ltd., Yokohama, Japan).** The transanal drainage system we used included a guide wire (white arrow, through-the-scope thin dilator, white arrowhead), and thick dilator (black arrow). The through-the-scope thin dilator had a blunt tip, white arrowhead). The guide wire, which was included in the system we used, had a soft flexible tip. By contrast, the tail of the guide wire was firm and can give a considerable penetrative force when thrust. Therefore, we inserted the guide wire backward into the through-the-scope thin dilator in order to reinforce the hardness of the through-the-scope thin dilator for moderate penetrative force.

**Figure 3:**
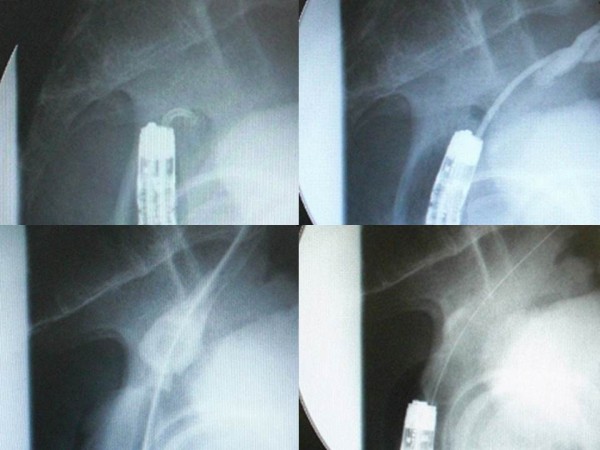
**Fluoroscopic findings of the procedure.** Firstly, we thrust the through-the-scope thin dilator, the hardness of which was reinforced with the reversely inserted guide wire, against the center of the circular staple line to penetrate the membranous structure (upper left). When the through-the-scope thin dilator was thrust, special attention was paid not to protrude the tip of the reversely inserted guide wire from the top of the through-the-scope thin dilator because the tail of guide wire was firm and relatively sharp, and it may have stuck to and injured other organs adjacent to the anastomosis if thrust in the wrong direction. We succeeded to penetrate the through-the-scope thin dilator through the membrane (upper right). Then, the guide wire was removed from the through-the-scope thin dilator to perform fluoroscopy via the through-the-scope thin dilator. Fluoroscopy confirmed that the tip of the through-the-scope thin dilator was present in the colon oral to the anastomosis (upper right). After that, the guide wire was inserted normally through the dilator and pushed orally as possible. The fiberscope was removed and a thick dilator was inserted over the through-the-scope thin dilator through the anastomosis under fluoroscopic guidance (lower left). After removing the dilators and guide wire, a colonoscope was reinserted to apply through-the-scope hydrostatic balloon dilatation (lower right).

**Figure 4:**
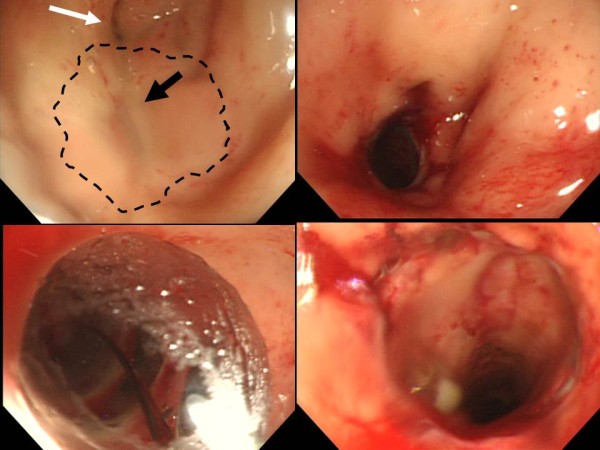
**Endoscopic appearance of the anastomotic ring.** Before the procedure, the anastomosis was completely obstructed. The expected location of the circular stapler ring is shown by dotted black line (upper left). The center of the circular stapler line was slightly depressed (black arrow, upper left). A single diverticulum was present near the anastomosis (white arrow, upper left). After penetration and dilatation by the guide wire and dilators, the anastomosis was opened (upper right) and easily facilitated the through-the-scope hydrostatic balloon dilator (lower left). After applying hydrostatic balloon dilatation, the fiberscope could easily go through the anastomosis (lower right).

A 79-year-old Japanese man underwent redo rectal resection for completely separated and obstructed anastomosis which was caused by anastomotic leakage after anterior resection. The initial surgery had been performed for advanced sigmoid colon cancer 3 years previously and the anastomotic leakage was remedied by diverging ileostomy performed 10 days after the initial surgery. Fluoroscopy conducted 1 week after the redo surgery showed good passage through the colorectal anastomosis and no anastomotic leakage. Ileostomy takedown was planned 6 months after the redo surgery for social reasons of the patient. Prior to the takedown, however, high-pressure fluoroscopy showed no passage of contrast medium through the anastomosis (Figure 1). Subsequent colonoscopy could not find any orifice of anastomosis connecting oral and anal intestines to the anastomosis. Thus complete obstruction of the colorectal anastomosis was diagnosed. Abdominal computed tomography confirmed that the colon oral to the anastomotic site and the rectum anal to the anastomotic site were connected at the anastomosis but a thick membranous structure was observed in the circular staple line (Figure 1). These findings suggested that patency of the anastomosis was discontinued by the thick membranous web composed in the circular stapler ring. Although one-more surgical revision was considered, we thought that further surgery should be avoided if possible because he had already undergone redo surgery for failed colorectal anastomosis and thus the next surgery would probably be more stressful than the prior surgery. It was obvious that some special techniques were required for treating this condition nonsurgically because any orifice which enables conventional balloon dilatation technique was not detectable at the anastomotic site. First of all, therefore, we applied a nonsurgical approach for treating this condition. In order to avoid thermal injury and sharp needle injury, we applied blunt penetration technique using a commercially available device for transanal ileus drainage for obstructing colorectal cancer (Transanal Ileus Tube®; Create Medic Co., Ltd., Yokohama, Japan; Figure 2). Before applying this technique, a detailed written informed consent was given to the patient. It clearly described the impracticality of other conventional nonsurgical approaches, off-label use of transanal drainage tube, and the possibility of bowel perforation caused by misdirected thrust. Furthermore, we informed him that emergent receliotomy might be needed if a misdirected thrust occurred. After that, he agreed to receive this technique and thus we performed the following procedures. Firstly, we used a through-the-scope thin dilator (TTSTD); its hardness was reinforced with a reversely inserted guide wire as a penetrator. We pushed it against the center of the circular staple line to penetrate the membranous structure (Figure 3). When the TTSTD was thrust, special attention was paid not to protrude the tip of the reversely inserted guide wire from the top of the TTSTD. Because the tail of the guide wire was firm and relatively sharp, it may have stuck to and injured other organs adjacent to the anastomosis if it protruded and thrust in the wrong direction. We succeeded to penetrate the TTSTD through the membrane (Figure 3). Then, the guide wire was removed from the TTSTD to perform fluoroscopy via the TTSTD which confirmed that the tip of the TTSTD was present in the colon oral to the anastomosis (Figure 3). If a misdirected thrust occurs and the fluoroscopy via the TTSTD shows small intestine or peritoneography other than the colon oral to the anastomosis, the procedure must be stopped and emergent celiotomy should be considered if the situation demands. Fortunately, however, the fluoroscopy showed that pushing the TTSTD was properly and successfully performed and thus we continued the procedure. After that, the guide wire was inserted normally through the dilator and pushed orally as possible. The fiberscope was removed and a thick dilator was inserted over the TTSTD through the anastomosis under fluoroscopic guidance (Figure 3). After removing the dilators and guide wire, a colonoscope was reinserted and found that the anastomosis was open but too stenotic to pass the scope through. Therefore we applied through-the-scope hydrostatic balloon dilatation (Figures 3 and 4). The successful dilatation facilitated passage of the colonoscope, allowing evaluation of the entire colon. Seven days after the procedure, confirmative colonoscopy was performed and showed that the anastomosis was patent but not stenotic. Two days after the confirmative colonoscopy, ileostomy takedown was performed. The patient is currently doing well and had no complaint arising from the anastomotic stricture 12 months after the ileostomy takedown.

## Discussion

There have been a few reports of successful nonsurgical treatment of completely obstructed anastomosis after rectal resection [2-5]. In these reports, specialized devices and/or methods for other particular purposes were utilized for the treatment: for example papillotomy knife [2], suprapapillary biliary puncture catheter [3], forward array echoendoscope [4], and endoscopic rendezvous technique requiring two scopes and two endoscopists [5]. These reported methods were carefully planned and successfully performed but required unintended usage of materials specialized for other particular purposes. Furthermore, electrocision and needle puncture might cause other organ injury adjacent to the anastomosis when these devices are pushed through in the wrong direction. The use of forward array echoendoscope and endoscopic rendezvous techniques are considered to be reliable methods to avoid misdirection. However, the former is usually unavailable because it requires a markedly particularized device which is not ubiquitous. The latter requires many hands and a complicated procedure. From these aspects, we believe that the blunt penetration technique we applied is superior to the above-stated methods in terms of the following advantages. First, this technique does not require any specialized instruments that only exist in tertiary medical centers. We only used a conventional colonoscope, transanal drainage system, and hydrostatic balloon dilatation system, all of which are available in most general hospitals. Secondly, the technique we applied is considered to be superior in safety to other methods utilizing electrocision or sharp needle puncture even if applied in the wrong direction. Nonsurgical treatment for completely obstructed anastomosis is necessarily performed almost always under fluoroscopic guidance not under direct vision. Therefore, misdirected thrust must be always considered an occasionally unavoidable complication. If it occurs, either electrocision or sharp needle puncture can easily injure other organs adjacent to the anastomosis. However, the TTSTD we thrust against the occluded anastomosis has a blunt tip and therefore is unlikely to stick to organs outside the intestine even if it is pushed through in the wrong direction.

## Conclusions

We recognize that the success rate of this blunt penetration technique may be low because we suppose there will be many occasions for which this technique is not effective or available: for example the membranous web composed in the anastomotic ring is too thick or firm to be penetrated by the blunt tip penetrator, or the anastomotic site is twisted or crooked. However, it can be tentatively tried because we consider that it hardly causes serious complication even if it results in failure.

## Consent

Written informed consent was obtained from the patient for publication of this case report and any accompanying images. A copy of the written consent is available for review by the Editor-in-Chief of this journal.

## Abbreviations

TTSTD: Through-the-scope thin dilator.

## Competing interests

The authors declare that they have no competing interests.

## Authors’ contributions

KY, DM, and YM performed surgery. KY, ST, and YM treated the patient early postoperatively. DM and ST took charge of outpatient clinic for this patient. KY and DM wrote the manuscript. CM, YM, and ST revised the manuscript for important intellectual content. All authors read and approved the final manuscript.
